# Adaptive Optics Optical Coherence Tomography Analysis of Induced Pluripotent Stem Cell-Derived Retinal Organoid Transplantation in Retinitis Pigmentosa

**DOI:** 10.7759/cureus.64962

**Published:** 2024-07-19

**Authors:** Masaharu Ishikura, Yuki Muraoka, Yasuhiko Hirami, Hung-Ya Tu, Michiko Mandai

**Affiliations:** 1 Department of Ophthalmology and Visual Sciences, Kyoto University, Kyoto, JPN; 2 Department of Ophthalmology, Kobe City Eye Hospital, Kobe, JPN; 3 Institute for Protein Research, Osaka University, Osaka, JPN; 4 Laboratory for Retinal Regeneration, RIKEN Center for Biosystems Dynamics Research, Kobe, JPN

**Keywords:** photoreceptor cells, adaptive optics, retinal cell transplantation, retina-like organoids, retinitis pigmentosa

## Abstract

This study evaluates the transplantation of induced pluripotent stem cell (iPSC)-derived retinal organoids into patients with advanced retinitis pigmentosa using adaptive optics optical coherence tomography (AO-OCT) to monitor retinal changes over two years post transplantation. Our results confirmed successful engraftment and increased retinal thickness, with AO-OCT providing detailed visualization of cellular structures such as an outer plexiform layer-like line and highly reflective particles within rosette-like formations, indicative of photoreceptor development. Immunohistological analysis in a parallel monkey model confirmed these structures as mature, functional photoreceptor rosettes. The integration of high-resolution AO-OCT with immunohistology provides critical insights into the structural and functional outcomes of transplantation and represents a promising advancement in the treatment of retinal degenerative diseases.

## Introduction

Induced pluripotent stem cell (iPSC) technology has emerged as a transformative breakthrough in regenerative medicine, providing a method for reprogramming mature somatic cells into a pluripotent state similar to that of embryonic stem cells (ESCs). This advancement not only circumvents the ethical issues associated with using ESCs but also opens new possibilities for treating a wide range of diseases. One of its most compelling applications is the creation of retina-like organoids by introducing specific differentiation-promoting factors that mimic the complex three-dimensional structure of the retina [1,2].

Transplantation of these retinal organoid sheets into animal models has yielded significant advances, notably in the formation of synaptic connections between transplanted photoreceptors and host bipolar cells, as well as signs of functional recovery [3]. Inspired by these successes in animal models, this approach has been extended to human patients with advanced retinitis pigmentosa. Recent observations in the two years following transplantation have shown promising results, including engraftment of the organoid sheets and increased retinal thickness [4]. However, conclusively identifying the differentiation of these cells into functional photoreceptors has remained challenging, primarily due to the limited resolution of conventional spectral-domain optical coherence tomography (SD-OCT).

To address this limitation, adaptive optics (AO) technology, originally developed for astronomical imaging to compensate for atmospheric distortions, has been repurposed for retinal imaging. By correcting for ocular aberrations, AO technology enables high-resolution imaging of the retina, capturing details at the cellular level [5]. Leveraging this AO technology, we have developed a prototype AO-OCT system. This novel implementation of AO-OCT allowed us to meticulously evaluate the retina’s neuroglial architecture with an unprecedented level of detail in both healthy subjects and those with epiretinal membranes [6].

In this study, we used AO-OCT to assess retinal morphology two years after transplantation of iPSC-derived retinal organoid sheets in a patient with advanced retinitis pigmentosa. Specifically, we confirmed the successful integration of these sheets into the host retina and assessed their potential for differentiation into mature photoreceptors.

## Technical report

This observational study and the clinical study involving the reported case adhered to the tenets of the Declaration of Helsinki. Written informed consent was obtained from the patient for all study procedures and examinations. The Institutional Review Board and Ethics Committee of Kyoto University Graduate School of Medicine (Kyoto, Japan) approved this observational study. The clinical study that involved the case in this manuscript was approved by the Certified Special Committee for Regenerative Medicine of Osaka University and the Health Science Council of the Ministry of Health, Labour and Welfare of Japan (jRCTa050200027).

This study involved a single patient diagnosed with retinitis pigmentosa who had received transplantation of iPSC-derived retinal organoid sheets at Kobe City Eye Hospital, Japan, two years and three months prior. The graft was located in the superotemporal region of the macula of the right eye.

The transplanted area was imaged using SD-OCT (Spectralis HRA+OCT; Heidelberg Engineering, Tokyo Japan), and scanning laser ophthalmoscopy (SLO) images were obtained using the OCT-A1 system (Canon Inc., Tokyo, Japan). We used a prototype AO-OCT system developed by Canon Inc., which features a deformable mirror for real-time correction of ocular aberrations and high wavefront correction efficiency. This system allows for the simultaneous acquisition of AO-OCT and SLO images with axial and lateral resolutions of 3.4 and 3.0 μm, respectively. Images of a 5° area centered on the fovea were acquired at a rate of 45 fps over 23 s for each retinal section. These images were processed using onboard software to reduce speckle noise and ensure accurate imaging of the retinal organoid sheet implantation area, previously identified using SD-OCT and AO-SLO. These imaging tests were conducted at Kyoto University.

Histological immunostaining data of the previously reported eight-year-old cynomolgus monkey eye (*Macaca fascicularis*) with human iPSC-derived retinal transplantation was compared for AO-OCT image interpretation [7]. All animals were treated in accordance with the Association for Research in Vision and Ophthalmology statement for the use of Animals in Ophthalmic and Vision Research. Animal experiments were conducted with the approval of the Animal Research Committee at RIKEN Center for Developmental Biology (now Center for Biosystems Dynamics Research). Briefly, the human iPSC-derived retina was differentiated from the human iPSC-1231A3 line (provided by Kyoto University) [8] according to the method described by Kuwahara et al. [9]. Photoreceptor cells were focally ablated using the 577-nm OPSL PASCAL laser system (Topcon Medical Laser Systems, Tokyo, Japan), and the hiPSC-retina (differentiation day 63) was transplanted in the outer nuclear layer (ONL) lost region approximately two months post-laser treatment. The eye of the cynomolgus monkey was then fixed with SUPERFIX (Kurabo), processed for paraffin embedding, and sectioned at 10-μm thickness. Our previous publication by Tu et al. described all the detailed procedures [7]. Sections were stained with the following antibodies: anti-rhodopsin (clone RET-P1, Sigma-Aldrich, 1:1000 dilution) and anti-recoverin rabbit (Merck Millipore, 1:1000 dilution) with nuclei counterstained with 4′,6-diamidino-2-phenylindole (DAPI, 1 μg/mL; Molecular Probes, Eugene, OR).

**Figure 1:**
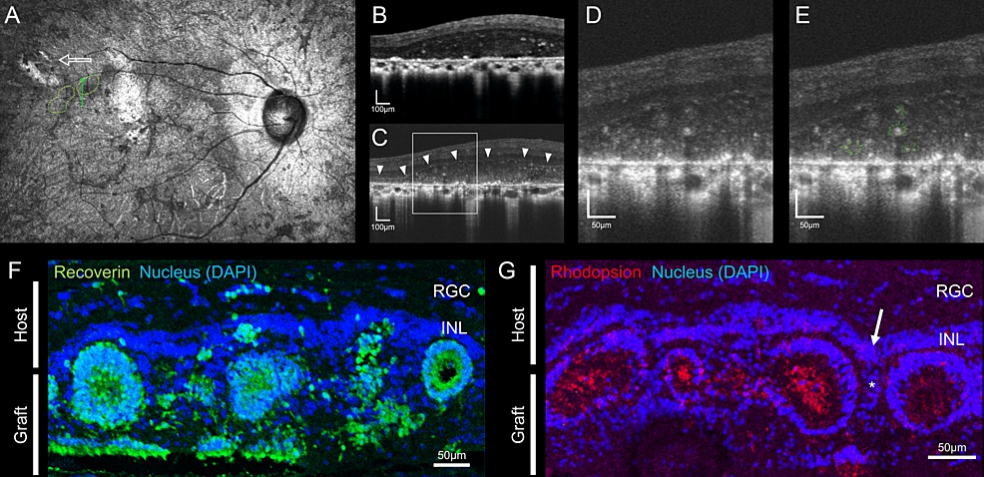
Photoreceptor and outer segment organization in rosette-like structures at the site of retinal organoid sheet transplantation and immunohistological images of human-induced pluripotent stem cell-derived retinas transplanted in a monkey model of laser-induced retinal degeneration A. Scanning laser ophthalmoscopy (SLO) image: White arrows indicate the incision for transplantation of the retinal organoid sheet, and the dotted line indicates the transplantation area. The green line indicates the spectral-domain OCT (SD-OCT) scan path. B. SD-OCT image of the transplantation site. A cross-sectional view highlighting structural changes after transplantation. C. Adaptive optics OCT (AO-OCT) images of the same site provide a high-resolution view detailing the outer plexiform layer (OPL) like line (arrowheads) and possible arrangement of the photoreceptor cells. D. Magnified AO-OCT image showing a detailed view of the rosette-like arrangement of the photoreceptor cells. E. Magnified AO-OCT image with color emphasis showing detailed photoreceptor structures (green) and their outer segments (yellow), illustrating the intricate architecture of the graft. F. Maturation of human iPSC-derived retinas in a monkey model of retinitis pigmentosa with laser photoreceptor ablation. A row of recoverin-positive transplanted photoreceptors are aligned with INL in the outer nuclear layer (ONL)-ablated region, suggesting a successful graft integration. G. Rhodopsin-positive transplanted photoreceptors show different degrees of outer segment development inside the photoreceptor rosettes, further confirming the maturation process and functional potential of the transplanted photoreceptor cells. Nuclear staining shows the presence of OPL-like spacing between the graft photoreceptors and host/graft INL. Graft INL (asterisk) is continuous from possibly merged host/graft INL (arrow). F-G. Both graft and host rod bipolar cells appear to coexist in an INL-like structure after iPSC-retinal organoid sheet integration, and an OPL-like space is formed, where bipolar cells form synapses with the graft photoreceptor cells.

The best-corrected visual acuity in the patient’s right eye was 0.07. Using SLO, we delineated the retinotomy site used for implanting the retinal organoids and accurately located the graft position within the retinal architecture (Figure 1A). Subsequent SD-OCT revealed rosette-like structures within the transplanted tissue (Figure 1B). With AO-OCT, the enhanced resolution of this technique provided a more detailed view, revealing an outer plexiform layer (OPL)-like line between the host inner nuclear layer (INL) and the graft area (arrowheads in Figure 1C). Additionally, the presence of similar multiple-round structures with a radial component was observed in the graft area. These round structures had varying degrees of hyper-reflective components inside (Figures 1D, 1E).

Further immune histological comparison in a monkey model of retinitis pigmentosa indicated the development of human iPSC-derived retinas in the ONL-ablated region in the monkey eye. The recoverin-positive-transplanted photoreceptors aligned to host INL suggest successful graft integration (Figure 1F). Additionally, these photoreceptors display varying degrees of rhodopsin-positive outer segment development within the photoreceptor rosettes, further validating the maturation process and the functional capacity of the transplanted cells (Figure 1G). Nuclear staining showed the OPL-like spacing between the INL structure and graft photoreceptor cells (Figure 1E). A careful observation reveals the merging point of graft (*) and host INL (arrow in Figure 1G). This may suggest the formation of an OPL-like space between the graft ONL and merged INL of host and graft, which may correspond to the OPL-like line observed by AO-OCT, where host bipolar cells may potentially form synaptic connections with the graft photoreceptor cells.

## Discussion

Maturation of graft photoreceptor cells is a major issue faced after iPSC-derived retinal organoid transplantation; however, monitoring the developing status of the graft is challenging. Previously, we reported that photoreceptors in transplanted retinal organoid sheets often form rosette structures with outer segment development inside them to different degrees and statuses, as shown in reference histology, which is considered a possible marker for estimating photoreceptor maturation. In this study, we used AO-OCT to evaluate retinal morphology in a patient with retinitis pigmentosa more than two years post transplantation of iPSC-derived retinal organoid sheets [4]. The superior resolution of AO-OCT revealed the presence of an OPL-like line and highly reflective particles within rosette-like structures, a detail not seen with conventional SD-OCT imaging.

These highly reflective particles exhibit characteristics consistent with the outer segment disks typically observed in healthy eyes using AO-OCT, suggesting that transplanted retinal organoid sheets may develop into functional photoreceptors. As previous animal studies have demonstrated light responsiveness in photoreceptor rosettes, our observations suggest that the transplanted area in this study may have functional potency.

Another feature better visualized using AO-OCT was the presence of OPL-like lines. Histologically, transplanted grafts formed the INL around the graft ONL rosettes, which were often observed to merge with the host INL [7,10]. In our previous observations, both human marker STEM 121 positive and negative rod bipolar cell bodies were observed in the INL. This indicates that the OPL-like line in AO-OCT, which lies between the INL and rosette-like structures in the graft area, may represent the synaptic site between the host/graft bipolar cells and graft photoreceptor cells. Another possibility is that the line represents the boundary of the differential reflectance between the host IPL-INL and non-aligned graft structures. In either case, the presence of OPL-like lines may indicate a preserved host INL after graft integration, which may support functional potency once host-graft synaptic connections are achieved.

Nevertheless, a cautious approach to interpreting these complicated images is required, given the current limitations in the resolution and technical capabilities of even the most sophisticated AO-OCT imaging. The challenge of accurately identifying the origins of various cellular components within the organoid sheets underscores the need for further research. Such investigations are essential to refine our methods and deepen our understanding, thereby improving our ability to accurately evaluate the results of retinal regeneration.

## Conclusions

As research advances, the application of noninvasive, high-resolution AO-OCT imaging is expected to become a fundamental aspect of retinal degeneration therapy. The continued refinement of imaging techniques, along with a better understanding of the dynamics of the retinal tissue after transplantation, holds promise for driving advancements in this field. These developments are critical for the successful clinical application of iPSC-derived retinal therapies, potentially leading to significant clinical breakthroughs based on the insights provided by AO-OCT.
